# Attentional suppression is in place before display onset

**DOI:** 10.3758/s13414-023-02704-6

**Published:** 2023-04-06

**Authors:** Changrun Huang, Mieke Donk, Jan Theeuwes

**Affiliations:** 1 Department of Experimental and Applied Psychology, Vrije Universiteit Amsterdam, Van der Boechorststraat 7-9, 1081 BT Amsterdam, The Netherlands; 2 Institute Brain and Behavior (iBBA), Amsterdam, the Netherlands; 3 William James Center for Research, ISPA-Instituto Universitario, Lisbon, Portugal

**Keywords:** Statistical learning, Visual selection, Distractor suppression

## Abstract

Recent studies have shown that observers can learn to suppress a location that is most likely to contain a distractor. The current study investigates whether the statistically learned suppression is already in place, before, or implemented exactly at the moment participants expect the display to appear. Participants performed a visual search task in which a distractor was presented more frequently at the high-probability location (HPL) in a search display. Occasionally, the search display was replaced by a probe display in which participants needed to detect a probe offset. The temporal relationship between the probe display and the search display was manipulated by varying the stimulus onset asynchronies (SOAs) in the probe task. In this way, the attentional distribution in space was probed before, exactly at, or after the moment when the search display was expected to be presented. The results showed a statistically learned suppression at the HPL, as evidenced by faster and more accurate search when a distractor was presented at this location. Crucially, irrespective of the SOA, probe detection was always slower at the HPL than at the low-probability locations, indicating that the spatial suppression induced by statistical learning is proactively implemented not just at the moment the display is expected, but prior to display onset. We conclude that statistical learning affects the weights within the priority map relatively early in time, well before the availability of the search display.

## Introduction

Humans are sensitive to spatial regularities in their environment. The experience with these regularities strongly biases the way in which people perform a visual search task. For instance, the repeated presence of road signs on the right side of the road biases observers to prioritize the selection of road signs located on the right over those located on the left side of the road. Past selection experience, i.e., selection history, has been suggested to be a major factor in the guidance of attention in visual search (Awh et al., 2012; Theeuwes, 2018, 2019).

Much research has been done to understand how attention is biased by spatial regularities in the location and context of a target (Geng & Behrmann, 2002, 2005; Jiang et al., 2013; Li et al., 2022; Li & Theeuwes, 2020). Recently, an increasing number of studies has focused on how spatial regularities in task-irrelevant but salient distractors affect visual search (Duncan & Theeuwes, 2020; Ferrante et al., 2018; Gao & Theeuwes, 2020; Goschy et al., 2014; Sauter et al., 2018, 2019, 2021; van Moorselaar et al., 2020, 2021; Wang & Theeuwes, 2018a, 2018b; Won et al., 2019; Xu et al., 2021). Typically, spatial regularities are introduced by presenting a salient yet irrelevant distractor more frequently at one location than at other locations. The typical finding is that visual search for a target is faster and more accurate when the distractor is presented at such a high-probability location compared to any of the other locations (Ferrante et al., 2018; Wang & Theeuwes, 2018a). To account for this finding, it has been proposed that through statistical learning, the frequent distractor location becomes suppressed relative to the other locations in the spatial priority map (Theeuwes, 2018, 2019; Theeuwes et al., 2022).

At present, it is unclear when distractor suppression induced by statistical learning is applied. One possibility is that suppression is applied in a reactive manner such that it occurs after attention has been captured by the irrelevant salient distractor. Statistical learning may then lead to faster suppression, allowing attention to be more rapidly disengaged from the distractor at the high-probability location as compared to the other locations. However, several studies showed that observers’ performance was not only enhanced when a distractor was presented at a high-probability location, but was also impaired when this location happened to contain the target (Ferrante et al., 2018; Huang, Theeuwes, & Donk, 2021a; Wang & Theeuwes, 2018a, 2018b, 2018c; but see Goschy et al., 2014; Liesefeld & Müller, 2021; Sauter et al., 2019). It seems unlikely that suppression is reactively applied since the target was suppressed when it was presented at a high-probability distractor location. Alternatively, suppression can be applied proactively, meaning that as a result of statistical learning, the frequent distractor location is suppressed prior to its selection. Note that this type of (learned) suppression is different from the suppression that has been proposed by the signal suppression hypothesis (Gaspelin et al., 2015, 2017; Sawaki & Luck, 2010, 2013). According to this account, suppression only operates on specific features, such as a specific color, and observers must have knowledge of the color they need to avoid. If suppression only operates on a specific feature such as the color red, then if the target is “not-red,” it should not be suppressed regardless of its location. If, however, suppression is location-based only, then regardless of its features, objects presented at these high-probability distractor locations should be suppression. Location-based suppression can explain why a target presented at a high-probability distractor location is suppressed even though it has features that are highly relevant to the task.

Indeed, several studies have been suggesting that the suppression of a frequent distractor location as induced by statistical learning occurs proactively (Huang et al., 2022; Huang, Theeuwes, & Donk, 2021a; Huang, Vilotijević, et al., 2021b; Wang, Samara, & Theeuwes, 2019a; Wang, van Driel, et al., 2019b). For example, in a study by Huang, Vilotijević, et al. (2021b), participants were asked to search for a unique shape that was presented simultaneously with an irrelevant colored distractor singleton and six neutral stimuli in a search display. The distractor was presented more frequently at one particular location to induce statistical learning. Occasionally, instead of a search display, a probe display was presented. Upon the presentation of the probe display, observers had to detect the offset of a probe dot, which occurred equally likely at each one of the eight possible locations. The probe display was used to provide a snapshot of the distribution of attention in space at the moment in time the search display was expected to be presented. The results showed that reaction times (RTs) to probe offsets presented at the high-probability distractor location were longer than those to probe offsets presented elsewhere. The observed difference in reaction times indicated that attention was directed away from the high-probability distractor location, suggesting proactive suppression of the high-probability distractor location in anticipation of the upcoming search display. Moreover, in a further study (Huang et al., 2022), it was shown that probe-offset detection performance was not only hampered at a high-probability distractor location but also simultaneously enhanced at a high-probability target location, suggesting that through statistical learning, both distractor suppression and target enhancement can occur simultaneously prior to attentional selection.

Although these findings showed that suppression was brought into force prior to display onset, the question remains whether distractor suppression is applied at the moment of onset of the search display or already prior to display onset. In Huang, Vilotijević, et al. (2021b) as well as in Huang et al. (2022), the probe display was always presented at the same moment in time as the search display was expected to occur. That is, both the probe display and the search display were presented 800 ms after the presentation of a placeholder display. The predictability of when the display would be presented may have allowed participants to only activate the suppression of the distractor location at the time when they expected the search display to appear. Accordingly, suppression may have been applied prior to attentional deployment but not prior to display onset. Indeed, in a recent study conducted by Grubert and Eimer (2020), it was shown that people were quite flexible in activating a specific attentional target template at the moment it became relevant (see also Xu et al., 2022). They showed that contingent upon the expected feature of an upcoming target, different attentional templates could be selectively activated. More importantly, a specific attentional template was not continuously activated but was rather re-activated over and over again around the time the search display was expected to be presented (see also Grubert & Eimer, 2018). It is possible that statistically learned suppression functions in a similar manner so that it is only activated at the time that observers expect the onset of the search display.

**Fig. 1:**
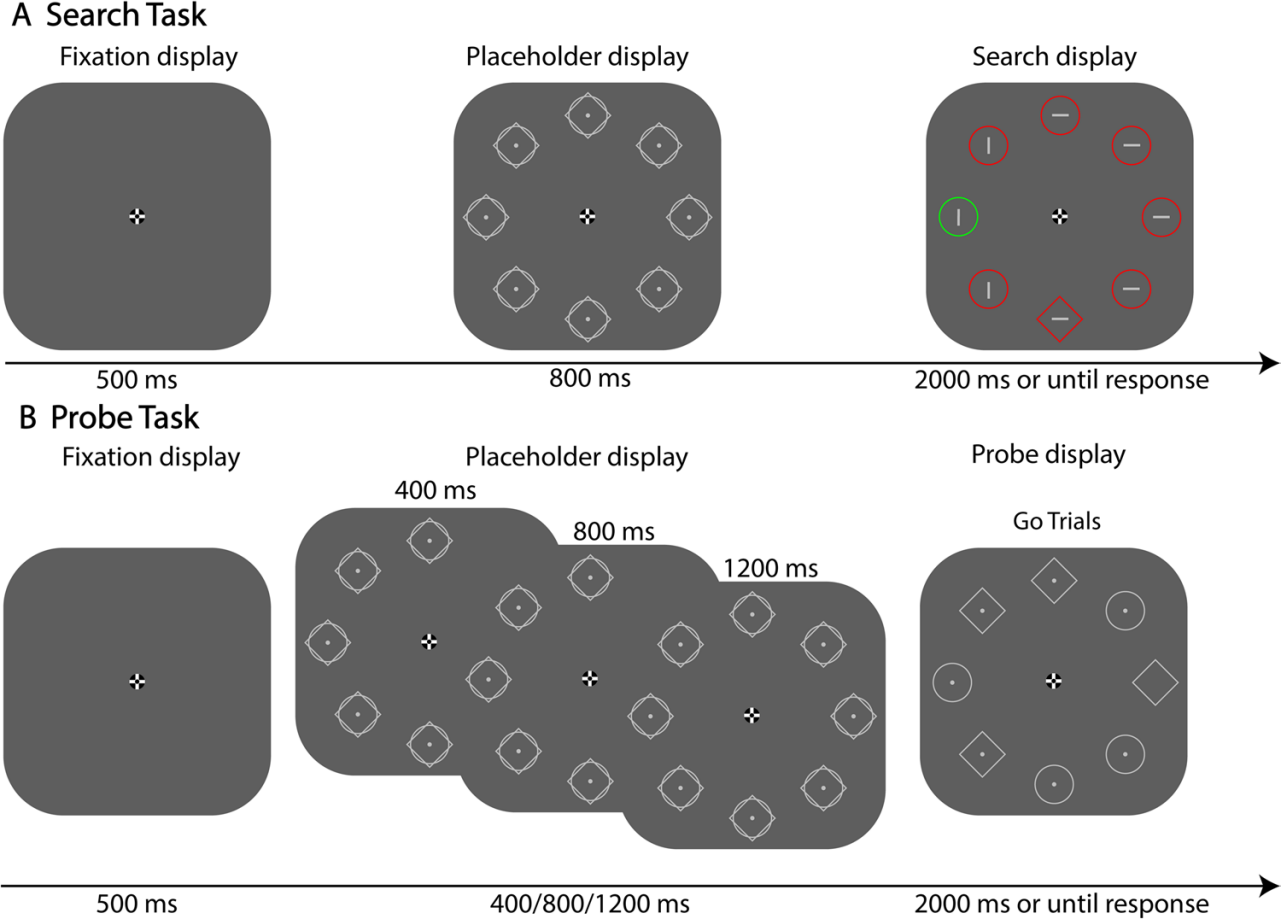
Example of the stimuli. **A** An example trial in the search task. Participants were asked to search for the target shape singleton (either a diamond amongst circles or a circle amongst diamonds) in the presence of an irrelevant distractor color singleton (either a green shape amongst red shapes or a red shape amongst green shapes). **B** An example trial in the probe task. Participants were asked to indicate the presence of a dot offset (Go trials) or refrain from responding if no dot was missing (No-Go trials)

The purpose of the current study was to examine whether distractor suppression is flexibly applied at the moment in time the onset is expected (see Grubert & Eimer, 2020) or whether it is applied during the time period before the display is presented. In the latter case, it is expected that the distractor location is already suppressed before the search display comes on. To distinguish between these two possibilities, we used a similar design as in our previous studies (Huang et al., 2022; Huang, Vilotijević, et al., 2021b) in which participants performed a search task on the majority of trials and a probe offset detection task in the remaining trials. The probe offset detection task is a method for evaluating how attention is allocated in space in a given display. It is inspired by the letter-probe task (Gaspelin et al., 2015; Kim & Cave, 1999), which is another commonly used technique for assessing the distribution of attention in space. In the search task, participants searched for a unique shape singleton in the presence of an irrelevant salient color distractor. To induce statistical learning, the distractor was presented more often at one particular location than at any of the other locations. In the probe offset detection task, the offset occurred equally likely at each of the locations. Crucially, the search display was presented consistently 800 ms after the presentation of a placeholder display, whereas the probe display could be presented at three different stimulus onset asynchronies (SOAs) relative to the placeholder display (400/800/1,200 ms, see Fig. 1). In this way, the probe display could appear prior to (in the 400-ms SOA condition), simultaneously with (in the 800-ms SOA condition), or after (in the 1,200-ms SOA condition) the expected onset of the search display. If suppression is flexibly applied and brought into force exactly at the onset of the search display, probe detection performance should only be affected by the distractor regularity at the 800-ms SOA, as this SOA is identical to the one used in the search task. If the high-probability distractor location is proactively and continuously suppressed, probe detection performance should be affected by the regularity, irrespective of SOA.

## Methods

### Participants

To determine the sample size, we ran an a priori power analysis using the *simr* package (Green & MacLeod, 2016) in an R environment (R Core Team, 2020). We took 19 ms as the effect size of interest based on the effect size (β = 18.87) reported in a study with a large sample size (N = 180) and a similar experimental design (Huang et al., 2022). The power analysis was performed using the data (N = 180) and the linear mixed model structure reported in Experiment 3 by Huang et al. (2022). The analysis indicated that a sample size of 72 participants would have a power of 80.7% (95% CI [78.11, 83.10] in 1,000 simulations) to detect a RT difference of 19 ms. We kept recruiting participants until the number of participants reached the predetermined sample size after data exclusion. In total, 75 participants (33 females, *M*_*age*_ = 24.6 years, *SD*_*age*_ = 4.3) were recruited via the Prolific platform. All participants received a monetary reward (£5.50) in exchange for 50 min of participation.

Before the experiment, all participants provided written informed consent. The experiment was approved by the Ethical Review Committee of the Faculty of Behavioral and Movement Sciences of Vrije Universiteit Amsterdam and was conducted in accordance with the guidelines of the Helsinki Declaration.

### Stimuli and task

The experiment was programmed in OpenSesame (Mathôt et al., 2012) using OSweb, and run on PC devices using JATOS (Lange et al., 2015). The sizes of stimuli are reported in pixels under a display resolution of 1,024 × 768 and the colors are reported in RGB values (red/green/blue). All stimuli were superimposed on a dark gray background (RGB: 94/94/94). The experiment comprised a *search task* in two-thirds of the trials and a *probe task* in one-third of the trials.

#### Search task

 We used a modified version of the additional singleton task (Theeuwes, 1991, 1992) as the search task. Each search trial started with a display showing a fixation dot for a duration of 500 ms. The fixation dot (20 × 20 px) remained on-screen throughout the search trial. Subsequently, a placeholder display was shown for 800 ms, followed by a search display for 2,000 ms or until a response was given. The placeholder display consisted of eight equidistant elements placed on an imaginary circle with a radius of 224 pixels around the central fixation dot. Each element was created by superimposing a diamond (115 × 115 px; RGB: 192/192/192) on a circle (102 × 102 px; RGB: 192/192/192) with a dot (9 × 9 px; RGB: 192/192/192) in the center. The search display consisted of one shape singleton (the target), one color singleton (the distractor), and six other non-singletons (see Fig. 1a). Each of them contained either a horizontal or vertical line (41 × 3 px; RGB: 192/192/192). The target could either be a diamond (115 × 115 px) amongst circles or a circle (102 × 102 px) amongst diamonds. The distractor was presented on each trial and was either colored red (RGB: 255/0/0) amongst green elements or green (RGB: 0/208/0) amongst red elements. Distractor color (red or green), target shape (circle or diamond), and line orientation within the target singleton (horizontal or vertical) were randomly determined on each search trial. Participants were instructed to search for the target and indicate the line orientation within it as fast and accurately as possible by pressing either the “up” or the “left” arrow key for vertical or horizontal orientation, respectively.

#### Probe task

 The probe task was similar to the search task except that the placeholder display was presented for 400, 800, or 1,200 ms (equally likely), followed by a probe display for 2,000 ms or until a response was given. The probe display consisted of four circles and four diamonds randomly distributed within the visual array. On 20% of all probe-task trials (No-Go trials), each shape contained a light gray dot in the middle, similar to the placeholder display. On 80% of all probe trials (Go Trials), one dot was missing in the probe display, creating a probe offset at that location relative to the placeholder display. Participants were instructed to press the spacebar key as fast as possible in trials with a probe offset (Go Trials) and withhold a response in trials without (No-Go trials).

### Design and procedure

To increase the statistical power for the probe task, only four locations were used to present the target and the distractor in the search task, and the probe offset in the probe task. These four locations (fixed for each participant) could either be at the diagonal positions or the horizontal and vertical positions of the imaginary circle (counterbalanced across participants). In the search task, one of these four locations represented a high-probability location (HPL), meaning that the distractor was more likely (65%) to be presented at that location than at any of the remaining three low-probability locations (LPLs). The target was equally likely presented at each of these four locations and never overlapped with the distractor. In the probe task, the probe offset occurred equally likely at each of these four locations.

The experiment consisted of 12 blocks of 90 trials. Each block comprised 60 search trials and 30 probe trials (six No-Go and 24 Go trials). In each block, the search trials and the probe trials were randomly intermixed with the constraint that two probe trials could not be presented in sequence. If participants made an incorrect response, a display with the letter ‘X’ was provided in the middle of the screen for a randomly varying duration between 800 and 1,000 ms. After each block, participants were given feedback about their average RTs and the percentage correct (calculated across trials regardless of trial types). Before the experiment, participants were given instructions and two practice blocks each to become familiar with both tasks. There was no distractor regularity during the practice blocks. Participants were only allowed to move on to the experiment if the accuracy in both practice blocks is above 55%. Two questions were used to assess participants’ awareness of the distractor regularity at the end of the experiment. Specifically, participants were asked if they were aware that one location contained the distractor more often than any of the other locations, and indicate which of the eight possible locations they thought contained the distractor more often regardless of their answer to the previous question.

### Data-analysis

For statistical analysis, we built generalized linear mixed models (GLMMs) on the accuracy data and linear mixed models (LMMs) on RTs using the *lme4* package (Bates et al., 2015) in R (R Core Team, 2020). The mixed-effect models have the advantage of handling the unbalanced design (as in the current study) and provide more statistical power to find the true effect by utilizing the dataset at the trial level (Brysbaert & Stevens, 2018).

For the search task, the distractor location (HPL, LPL) was included in the fixed-effects structure as the factor of interest. To ensure that distractor location (HPL, LPL) is not confounded with target location, we excluded trials in which the target was presented at the HPL. To control the variance that may be explained by the irrelevant features of the task or the stimuli, we included the following control factors in the fixed-effects structure: target line orientation (horizontal, vertical), target shape (circle, diamond), target color (green, red), the physical locations of the target (0~7), the physical locations of the distractor (0~7), target location priming (yes, no), distractor location priming (yes, no), probe-target location priming (yes, no), probe-distractor location priming (yes, no), and distractor awareness (yes, no). Here, location priming refers to whether a specific item was presented at the same location in consecutive trials. The random-effect structure was determined by running the maximal effect structure justified by the design (Barr et al., 2013). Specifically, the random-effects structure included by-participants random intercepts and by-participants random slopes for distractor location. The LMMs of RTs and the GLMMs of the accuracy data shared the same fixed- and random-effects structure. Additional analyses were run to test whether target processing was hampered when the target was presented at the HPL. The mixed-effect models were built by using the same fixed- and random-effects structure as mentioned above but replacing the factor of interest (i.e., distractor location) with target location (HPL, LPL). To prevent target location (HPL, LPL) being confounded with distractor location, we excluded trials in which the distractor was presented at the HPL in this latter analysis.

For the probe task, SOA (400, 800, 1,200 ms), probe location (HPL, LPL), and their interaction were entered into the fixed-effect structure as the factors of interest. Other control factors in the fixed-effects structure included physical probe location (0~7), target-probe location priming (yes, no), distractor-probe location priming (yes, no), and distractor awareness (yes, no). By-participants random intercepts and by-participants random slopes for probe location were included as random effects. All fixed effects were dummy coded. The degrees of freedom were estimated by the Satterthwaite approximation and the p-values were obtained from the *lmerTest* package (Kuznetsova et al., 2017). The Bayes factors (BF01) favoring the null hypothesis were calculated and reported for insignificant findings using *BayesFactor* R package (Morey et al., 2018) with the default prior (Jeffreys-Zellner-Siow, JZS). The estimate (β) of each fixed effect of interest was provided as the measure of the effect size.

## Results

In total, three participants whose accuracy was lower than 2.5 standard deviations of the overall mean accuracy in the search task were excluded, leaving 72 participants for analysis. Incorrect responses, as well as responses that were faster than 200 ms, were excluded from the RT analyses. For each participant, the trials with the RTs that exceeded the ±2.5 standard deviation from the overall mean RT (collapsed across conditions) were also removed from the RT analyses.

### Search task

**Fig. 2:**
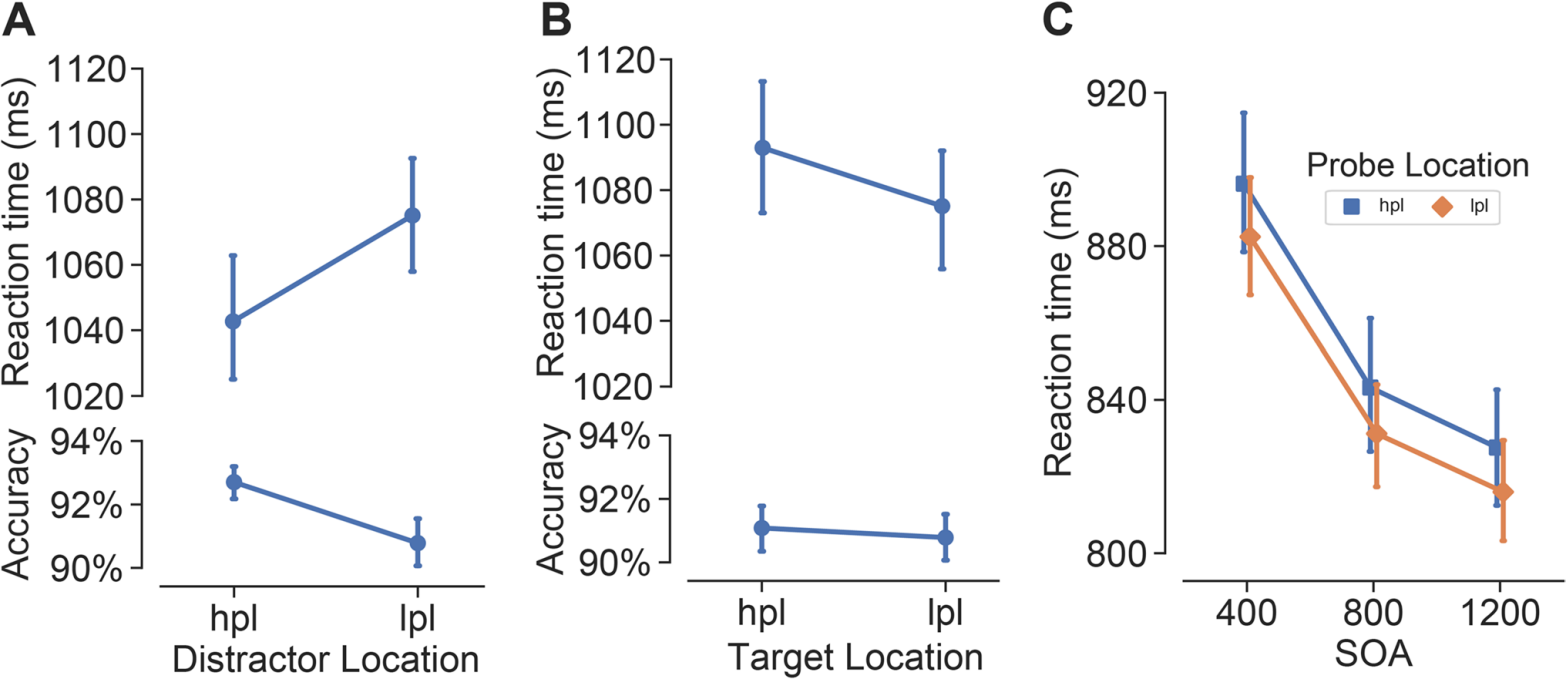
Statistical learning (SL) effects. **A** Mean reaction times (RTs) and accuracy in the search task when the distractor was presented at the high-probability location (HPL) or at a low-probability location (LPL). **B** Mean RTs and accuracy in the search task when the target was presented at the HPL location or at a LPL (indirect effect of SL). **c** Mean RTs as a function of probe dot location (HPL, LPL) in the probe task. Error bars denote ±1 *SE*_*mean*_

 As shown in Fig. 2a, responses were faster (β = 31.4, *SE* = 4.15, *t*(75.1) = 7.57, *p <* .001) and more accurate (β = 0.24, *SE* = 0.065, *z* = 3.67, *p* < .001) when the distractor was presented at the HPL than at the LPLs, indicating that the distractor was suppressed for attentional selection when it was presented at the HPL. Moreover, the target search was slower (β = 18.1, *SE* = 5.59, *t*(68.7) = 3.24, *p =* .002) when the target was presented at the HPL as compared to the LPLs (see Fig. 2b). No significant difference was found in the search accuracy between trials in which the target was presented at the HPL and the LPLs (β = 0.04, *SE* = 0.069, *z* = 0.65, *p* = .518, BF01 = 28.79). Together, these results suggested that statistical learning yielded a feature-blind spatial suppression on the HPL so that any singleton (target or distractor) presenting at this location would compete for less attentional selection.

### Probe task

 In general, participants performed well in the probe task. They had low false alarm rates in the No-Go trials (M = 6.3%, SD = .048) and low miss rates for the Go trials (M_HPL_ =1.1%, SD_HPL_ = .028, M_LPL_ = 0.9%, SD_LPL_ = .015).

Figure 2c shows the mean RTs as a function of probe location (HPL, LPL) and SOA (400, 800, 1,200) in the probe task. The LMMs analysis on RTs revealed a significant main effect of probe location (χ^2^(1) = 3.98, one-tailed *p =* .023). Participants were slower at detecting the probe offset at the HPL than at the LPLs (β = 14.6, *SE* = 7.24, *t*(86) = 2.02, *p* = .046). There was also a significant main effect of SOA (χ^2^(2) = 0.51, *p <* .001), with faster probe detection at 1,200 ms SOA than at 800 ms SOA (β = 17.3, *SE* = 3.49, *t*(20402) = 4.95, *p <* .001), and at 800 ms SOA than at 400 ms SOA (β = 64.4, *SE* = 3.49, *t*(20402) = 18.43, *p <* .001). However, the interaction between probe location and SOA was not significant (χ^2^(2) = 0.51, *p =* 0.773). The Bayes factor analysis suggested extreme evidence (BF01 = 517.40) for the model without the interaction effect (null hypothesis) relative to the model with it (alternative hypothesis).

### Awareness test

 Thirty-one participants reported that they were aware of the HPL during the experiment, and 18 of them correctly indicated the HPL. None of the remaining 41 participants, who reported being unaware of the HPL, correctly indicated the HPL. Several model comparisons were run on RTs and/or accuracy data to check whether awareness (yes, no) interacted with distractor location (HPL, LPL) or target location (HPL, LPL) in the search task, and probe location (HPL, LPL) in the probe task. Participants were labeled as ‘yes’ in the awareness factor if they reported being aware and correctly indicated the HPL. Plan model-comparisons in the search task indicated no interaction between awareness and distractor location in RTs (χ^2^(1) = 1.47, *p =* .226, BF01 = 10.17) and accuracy (χ^2^(1) = 2.48, *p =* .115, BF01 = 14.34), and no interaction between awareness and target location in RTs (χ^2^(1) = 0.81, *p =* .367, BF01 = 6.42) and accuracy (χ^2^(1) = 0.01, *p =* .918, BF01 = 17.94). Planned-model comparisons in the probe task also indicated no significant interaction between awareness and probe location in RTs (χ^2^(1) = 0.54, *p =* .463, BF01 = 12.50).

## Discussion

The current study investigates whether spatial suppression induced by statistical learning is applied at the learned (expected) time of display onset or prior to display onset. To this end, we combined an occasional probe offset detection task with a search task in which a distractor was presented more frequently at one location to induce statistical learning. Crucially, we manipulated the temporal relationship between the probe display and the search display such that the probe display could be presented before, simultaneously with, or after the expected onset of the search display. We replicated the classic statistical learning effect of Wang and Theeuwes (2018a) by showing a facilitated search for the target when the distractor was presented at the HPL (see also Ferrante et al., 2018). As shown before, target search was hampered when it was presented at the HPL, indicating that the suppression was spatial and basically feature-blind.

The critical finding of the current study is that regardless of the SOA, probe detection was always slower when the probe offset occurred at the HPL as compared to the LPLs. This indicates that prior to display onset, there was already suppression of the location where during the search the distractor was likely to be presented. It is important to note that in two-thirds of the trials, participants performed the visual search task in which the search display was revealed exactly after an SOA of 800 ms. If suppression would have been applied only at the moment the search display was expected to appear, one would have expected attentional suppression to only occur at an SOA of 800 ms as participants only had experience with this SOA during visual search. Clearly, our data indicated no hint of interaction between SOA and probe location, suggesting that suppression was already in place well before the display came on.

Visual search is hypothesized to be guided by a spatial priority map where the location with the highest weight will be prioritized for attentional selection. Recent progress in the field of visual attention has recognized that the priority map is formed by integrating the influences of goal-driven, salience-driven, and history-driven (e.g., statistical learning, value, and priming) factors (Awh et al., 2012; Theeuwes, 2019; Theeuwes et al., 2022; Wolfe, 2021). The current findings suggest that prior to display onset, the spatial priority map as it is shaped by statistical learning is already in place deprioritizing the location that is likely to contain the distractor. After display onset, the priority map is further updated by integrating the statistical learning effect with the influences of top-down and bottom-up effects. This is consistent with the findings of Gao and Theeuwes (2020), who showed that biases due to statistical learning and biases due to explicit top-down instructions independently affected visual selection, suggesting that these effects independently modulate the activity within the spatial priority map. Similarly, Huang, Theeuwes, and Donk (2021a) showed that eye movements were driven by the integrated spatial weights calculated based on the contributions of salience-driven, goal-driven, and selection history. Critically, the earliest possible oculomotor responses were already biased away from the high-probability distractor location, suggesting that this bias was already in place before the first eye movement was launched.

Unlike the findings of Grubert and Eimer (2020), the statistically learned suppression in the current study was not implemented only at the moment when the search display was expected. Instead, the results from the probe task suggested that the suppression was also applied 400 ms before and after the expected display onset. This inconsistency can be attributed to the different underlying processes. In Grubert and Eimer (2020), participants were explicitly informed of the alternating nature of the target. Therefore, it was possible for them to volitionally activate the relevant target template through top-down control. In contrast, in the current study, the spatial regularity of the distractor in the current study was unbeknown to participants and could only be learned through repetitive experiences.

The findings from the current study are to be distinguished from the proactive suppression process proposed by the signal suppression hypothesis (Gaspelin et al., 2015, 2017; Sawaki & Luck, 2010, 2013). The hypothesis posits that briefly *after* the presentation of the search display, the irrelevant but salient singleton will automatically generate an attend-to-me signal that aims to capture attention. Such an attend-to-me signal can be proactively suppressed before any attentional orientation has been made. In this sense, according to Gaspelin et al. (2015), proactive suppression does not mean *before* display onset (as we suggest here), but instead implies suppression before attention has shifted to the location. Notably, the suppression process as adhered to by the signal suppression hypothesis is assumed to be feature-based and engaged in a search scenario that required a feature search mode (Bacon & Egeth, 1994). In contrast, the task employed here encourages the use of what has been called the “singleton-detection mode,” and as such, there is no room for feature-based suppression as described by the signal suppression hypothesis (Gaspelin et al., 2015, 2017; Sawaki & Luck, 2010, 2013). Nevertheless, previous studies have shown that the type of learned suppression that we investigate here also occurred when participants engaged in feature search (van Moorselaar et al., 2021; Wang & Theeuwes, 2018c), indicating that learned suppression is not limited to any particular search strategy.

Although the purpose of the current study is to elucidate the proactive aspect of the statistically learned suppression, it does not necessarily suggest that proactive and reactive mechanisms are “all-or-none.” In fact, there is ample evidence that the reactive mechanism also plays a role in addition to proactive suppression. Several eye-tracking studies have indicated the co-existence of both mechanisms (Huang, Theeuwes, & Donk, 2021a;Sauter et al., 2021 ; Wang, Samara, & Theeuwes, 2019a). While observers were preparing for the upcoming visual search, the suppression was proactively implemented at the high-probability location so that fewer initial saccades landed on the distractor at that location. Occasionally when the eye movements were captured by the distractor, a rapid disengagement of the eyes was found when the distractor was presented at the high-probability location (Sauter et al., 2021; Wang, Samara, & Theeuwes, 2019a), suggesting a post-capture suppression process. Note that the post-capture suppression could have also been considered as a sustained effect or a “leak” from proactive suppression as the rapid disengagement from the distractor at the high-probability location has been found even in the earliest possible initial saccades (Sauter et al., 2021), suggesting that such eye-movements have been programmed beforehand.

One question that the current study cannot answer is whether the suppression is constantly present throughout the experiment or whether it is brought into operation each trial, during the inter-stimulus interval in the anticipation of the upcoming search display. Using EEG recording, Wang, van Driel, et al. (2019b) showed that starting at about 1,220 ms before display onset, the high-probability location was suppressed as evidenced by increased parieto-occipital alpha power contralateral to the high-probability distractor location. This suggests that suppression is not in operation throughout the experiment but is applied in each trial well before the anticipated display onset.

The current experiment shows that suppression is not only applied at the moment in time the search display is presented. Indeed, 400 ms before the search display comes on, there is already suppression of a location within the placeholder display that, during a search display, typically contains a distractor. The current study using the probe technique cannot answer the question of whether there is also suppression of the anticipated high-probability location in a completely empty field. To measure the distribution of attention before the search display appears, we need a placeholder display. A recent study has shown that the presence of any stimulus may prompt the retrieval of the assumed priority map with its learned weights (see Duncan et al., 2022).

In sum, the present results indicated that the spatial suppression induced by statistical learning was proactively implemented not just before attentional orientation but also prior to the expected onset of the search display. We conclude that statistical learning exerts its influence on the first feed-forward information that is utilized to shape the priority map at a relatively early point in time.
